# Gene Silencing via Ingestion of Double-Stranded RNA in Wireworm of *Agriotes* Species

**DOI:** 10.3390/insects15120983

**Published:** 2024-12-11

**Authors:** Jyoti Joshi, Robert Coffin, Ryan Barrett, Gefu Wang-Pruski

**Affiliations:** 1 Faculty of Agriculture, Dalhousie University, Truro, NS B2N 5E3, Canada; jyoti.joshi@dal.ca; 2 Privar Farm Inc., Quinte West, ON K8V 5P6, Canada; coffinpotato@gmail.com; 3 Prince Edward Island Potato Board, Charlottetown, PE C1E 2C6, Canada; ryan@peipotato.org

**Keywords:** wireworm, click beetle, *Agriotes* species, RNA interference, target genes, dsRNA delivery, pest control

## Abstract

Wireworms are the most destructive soil insect pests affecting horticultural crops. The damage often renders them unsuitable for commercial purposes, resulting in substantial economic losses. RNA interference (RNAi) has been broadly used to inhibit gene functions to control insect populations. It employs double-stranded RNA (dsRNA) to knockdown essential genes in target organisms, rendering them incapable of development or survival. Although it is a robust approach, the primary challenges are identifying effective target genes and delivering their dsRNA into wireworms. Thus, the present study established a liquid ingestion methodology that efficiently delivers dsRNA into wireworms. We then investigated the effects of four target genes on wireworm mortality. The highest mortality rate reached 50% when the gene encoding vacuolar ATPase subunit A was targeted. Its transcript content in the fed wireworms was also significantly reduced. The mortality rates of the other three target genes of vacuolar ATPase subunit E, beta-actin, and chitin synthase 1 were 28%, 33%, and 35%, respectively. This is the first report demonstrating an efficient feeding methodology and the silencing of target genes in wireworms. Our findings indicate that RNAi is an effective alternative method for controlling the wireworm pest, and can be used to develop field treatment strategies.

## Simple Summary

Wireworms, the larvae of click beetles (*Agriotes* species), are notorious insect pests that damage crops worldwide and cause huge economic losses. Several studies have attempted to identify alternate management strategies, including the use of pesticides. As a biotechnological alternative, RNA interference (RNAi) has been used to control insect pests. RNAi can prevent an organism from developing or surviving by knocking down genes in the target organism. RNAi-based control approaches can be highly species-specific due to their sequence-dependent nature and their use of the naturally occurring biological molecule of double-stranded RNA (dsRNA). However, the lack of genomic information on wireworm of *Agriotes* species makes it challenging to identify effective target genes and subsequently deliver their dsRNA into wireworms. To this aim, we developed a feeding method to deliver exogenous dsRNA to wireworms and then examined four target genes on mortality. The results indicated that the dsRNA from one target gene ingested by wireworms caused 50% mortality relative to the control, whereas the other three target genes resulted in 28% to 35% mortality. The RT-qPCR method also confirmed that the treated wireworms had lower gene expression levels. Overall, this study provides valuable insights for developing dsRNA-based insecticides against wireworms.

## 1. Introduction

Wireworms are the soil-dwelling larvae of *Agriotes* species (Elateridae; Coleoptera; common name: click beetle). They are phytophagous insects known as one of the most severe and widespread agricultural pests worldwide [1]. Wireworms damage crops by feeding on the seeds, roots, or lower stems of almost all agricultural crops, such as potatoes, carrots, strawberries, cabbages, lettuce, corn, and other cereal crops, resulting in yield reduction and reduced quality of crops [1]. The biology and ecology of some *Agriotes* species have been thoroughly studied [2,3,4,5], but it is difficult to distinguish these species based on morphological characteristics at the larval stage. Also, the life cycle of many *Agriotes* species using instar has not been clarified [1]. It is understood that wireworms have high sustainability and a multi-annual life cycle, living up to three to five years in the soil before emerging in the spring as mature click beetles [6]. Three destructive European species, *Agriotes obscurus*, *A. lineatus*, and *A. sputator*, have come to dominate in Atlantic Canada over the past ten years, particularly affecting potato crops [1]. The difficulty in determining wireworms using morphological features has prompted the introduction of molecular markers. For example, the mitochondrial cytochrome oxidase I (mtCOI) and 16S ribosomal RNA (16S rRNA) genes have been used for wireworm identification [7,8].

Several methods have been reported to control wireworm damage in Canada. One is by utilizing attractive beetle traps, such as the Vernon pitfall pheromone trap using species-specific pheromones [9] and the Noronha Elaterid Light Trap using a solar-powered spotlight [10]. These traps catch adult click beetles from fields, thereby indirectly reducing wireworm propagation. However, these traps can only be utilized for a limited period, such as April to June, when click beetles are active and seeking out food. They die out once they mate. Since wireworms thrive in the soil for several years, mass trapping is an ineffective strategy for controlling their population. Another approach is to use insecticides, such as organochlorines, organophosphates, phenylpyrazols, pyrethroids, and neonicotinoids, to combat wireworms. These have demonstrated partial success [1]. However, some of these chemicals are under re-evaluation or are scheduled for deregistration by the Pest Management Regulatory Agency (PMRA) [1] for environmental and health concerns. Recently, a new insecticide has been registered, Cimegra (BASF Canada Agricultural Solutions), which facilitates the control of prevalent and difficult-to-control chewing insects, including wireworms and corn rootworms [11]. However, this pesticide is not environmentally safe as it is toxic to other species, such as bees, fish, and aquatic invertebrates [12]. Although soil fumigation is an effective approach in some regions, Prince Edward Island (P.E.I.), which is Canada’s largest potato-producing province, has prohibited the practice; likewise, broad-spectrum soil fumigation is becoming less frequently used in many other countries due to soil health and environmental concerns. Biopesticides, such as entomopathogenic fungi (*Metarhizium* spp.), have also been observed to infect and kill wireworms [13,14]. However, they have not yet been demonstrated to work commercially because of their low efficiency and unmanageability in field applications. Rotation crops, such as brown mustard (*Brassica juncea*) and buckwheat (*Fagopyrum esculentum*), have shown promise in controlling wireworms in soil [15], but they do not give consistent results from year to year and often require sacrificing a commercial crop in the rotation year, along with soil disturbance.

In recent years, double-stranded RNA (dsRNA)-mediated RNA interference (RNAi) technology [16,17] has proven to be an efficient tool for controlling insect pests in a species-specific manner [18,19,20,21,22]. RNAi is a highly conserved sequence-specific method that inhibits the expression of a targeted gene in a target organism, inhibiting its growth and development with no deleterious effects on other organisms. To date, only one product, the transgenic GM-maize SmartStax PRO expressing dsRNA targeting the *Dv-snf7* gene in the Western corn rootworm, *Diabrotica virgifera virgifera*, has been approved in Canada (2016) and the United States of America (2017) [23]. SmartStax PRO presents a breakthrough in using RNAi technology in agriculture [23]. Another transgenic product based on the RNAi-mediated knockdown of the *Dv-SSJ1* gene has been tested [24,25].

To use RNAi technology, finding effective target genes (proteins) essential to an organism is key. To date, very little information has been published on wireworm genomics. This study identified four target genes using the genome information of closely related species, allowing us to design an RNAi procedure to control wireworm pest populations effectively. The four target genes selected for this study were vacuolar ATPase subunit A (V-ATPase A), vacuolar ATPase subunit E (V-ATPase E), chitin synthase 1 (CHS1), and beta-actin. Vacuolar-type H^+^-ATPase (V-ATPase), one of the most essential enzymes present in almost all eukaryotic cells, is responsible for generating energy gradients in many membranes and organelles [26,27,28]. It is an evolutionarily conserved muti-subunit enzyme that consists of two domains (V_1_ and V_O_ complexes). The V_1_ complex comprises eight subunits (A_3_, B_3_, C, D, E_3_, F, G_3_, and H) present in different copy numbers and hydrolyses ATP to provide energy for the translocation of protons across membranes [29]. Most functional RNAi studies on insect V-ATPase subunits have focused on the genes encoding V_1_ subunits A and E. High mortality has been reported in Coleoptera by silencing the gene expression of V-ATPase subunits A and E [18,30], and moderate mortality has also been reported in Hemiptera, Lepidoptera, Diptera, and Hymenoptera. Chitin synthase (CHS) catalyses chitin synthesis, a critical structural component of insect exoskeletons [31]. Generally, two distinct chitin synthase genes, CHS1 and CHS2, are identified in most insects. CHS1 is specifically responsible for the synthesis of chitin in the epidermis, trachea, eggs, and eggshell, whereas CHS2 is exclusively expressed in the midgut epidermal cells involved in the hydrolysis of the peritrophic matrix of insects [31]. The beta-actin gene is involved in various biological activities, such as muscle contraction, cell motility, cell division, and cytokinesis [32]. These four selected target genes have been extensively studied for their effects on the viability, growth, development, and reproduction of insect pests [18,33,34,35,36,37].

The success of RNAi depends on a sufficient amount of dsRNA being delivered to the target organism. Methods such as microinjection, feeding through an artificial diet, and soaking in dsRNA solution have been used on other insect species [38,39,40]. However, none could be directly employed for wireworms due to their small and fragile body sizes, unavailability of an artificial diet for feeding, and high mortality with soaking procedures. Hence, we developed a liquid ingestion methodology to deliver exogenous dsRNA molecules into wireworms. We also tested the efficiency of the RNAi procedure based on four target genes and their effect on wireworm mortality. Our findings provide effective candidate targets to control wireworm populations in agricultural fields.

## 2. Materials and Methods

### 2.1. Sampling and Rearing of Wireworms in the Laboratory

**Figure 1:**
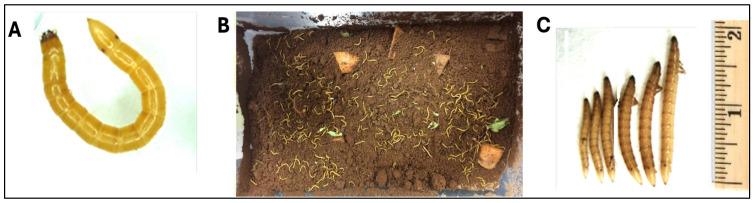
Rearing of wireworms. (**A**) Wireworm larvae of Agriotes species (click beetle). (**B**) Wireworms were maintained in a container with moist soil and pieces of carrot as food. (**C**) Different sizes of wireworms were used for the liquid ingestion assays. Images were taken by an iPhone and stereomicroscope (Leica, MZFIII, Leica Microsystems Ltd., Heerbrugg, Switzerland).

Wireworms (*Agriotes* spp.) were collected from potato, carrot, and cabbage fields near Charlottetown, P.E.I., and Truro, Nova Scotia, Canada. The wireworms were trapped using pieces of carrot or potato [41] from the uncultivated fields as bait. Baits were retrieved after 5–6 days and the wireworms were collected with the help of a sieve (1.0 mm sieve size) to remove rocks, roots, and other coarse organic matter. Also, we collected wireworms directly from an infested cabbage field. The rearing of wireworms in the laboratory took place in moist soil (~5 cm deep) with pieces of potatoes or carrots in a well-aerated container (16 cm × 13 cm × 8 cm) (Figure 1). To avoid the overcrowding of wireworms, approximately 300 worms were kept in each container. Wireworms were kept in a dark growth chamber at 22 ± 2 °C (room temperature, RT) until further use. To provide a satisfactory growth environment, the soil’s moisture level was regularly maintained (60% relative humidity), and the food was replaced weekly. Wireworms were also stored in the fridge at a lower temperature (6–8 °C) with food for more prolonged larval survival until further use. Two days before the date of the feeding experiment, the wireworms that were stored in the cold (6–8 °C) were returned to RT and kept in the dark.

### 2.2. Genomic DNA Extraction, PCR Amplification, and Sequencing of mtCOI Gene

To identify the Agriotes spp. in a given agricultural field, some wireworms were randomly selected for genomic DNA extraction using the cetyltrimethylammonium bromide (CTAB) method [42] with some modifications. Briefly, larvae were thoroughly pulverized in liquid nitrogen in a 1.5 mL microcentrifuge tube. A total of 450 μL of TES buffer (100 mM Tris HCl, 10 mM EDTA, 2 % SDS) and Proteinase K (20 μL, 10 mg/mL) were added, and samples were incubated overnight at 56 °C. The next day, 150 µL of 5 M NaCl and 1/10 vol of 10% CTAB were added, followed by incubation at 65 °C for 10 min. Then, 1 volume of chloroform:isoamyl alcohol, 24:1 was added, mixed gently and incubated for 30 min on ice. Samples were centrifuged at 20,000× *g* for 10 min at 4 °C and, the supernatant was then transferred to a new 1.5 mL microcentrifuge tube. Next, 225 µL of 5 M sodium acetate was added, mixed gently and placed on ice for 60 min. Later, the samples were centrifuged for 20 min at 21,000× *g* at 4 °C and the supernatant was transferred to a new 1.5 mL microcentrifuge tube. Genomic DNA was precipitated by adding 0.5 vol of isopropanol and incubating the samples overnight at 4 °C. The next day, the samples were centrifuged at 13,000× *g* at 4 °C for 10 min. The resulting DNA pellet was washed twice with 500 μL of 70% ethanol (*v*/*v*), air-dried for 30 min, then dissolved in 50–100 µL of Tris-EDTA (TE) buffer (10 mM Tris-Cl and 1 mM EDTA). Finally, 1 µL of RNase (10 µg/µL, NEB) was added and samples were stored at −20 °C until use.

After extraction, the partial mtCOI gene (710 bp) was amplified using PCR with universal invertebrate primers LCO1490 (5′-GGTCAACAAATCATAAAGATATTGG-3′) and HCO2198 (5′-TAAACTTCAGGGTGACCAAAAAATCA-3′) [43]. Then, PCR products were purified using the GenElute PCR Clean-Up kit (MilliporeSigma Canada Ltd., Oakville, ON, Canada), as per the manufacturer’s instructions, and DNA was sequenced at the Faculty of Agriculture, Dalhousie University, Canada. The DNA sequences were subjected to National Centre for Biotechnology Information (NCBI) BLAST database to confirm the mtCOI gene with reported species, and then the raw sequences were edited and aligned to a reference sequence of the mtCOI gene (NCBI accession numbers: KR124576.1 (*A. sputator*); KJ966025.1 (*A. lineatus*); and KM442197.1 (*A. obscurus*) using ClustalW (Bioedit)) [44].

### 2.3. Selection and Sequencing of Target Genes

In this study, four target genes were selected as RNAi targets: V-ATPase A, V-ATPase E, CHS1, and beta-actin. These target genes were selected based on previous reports of mortality in other insect populations exposed to the dsRNA of V-ATPase A [18,45], V-ATPase E [18], CHS1 [31], and beta-actin [46]. Since the wireworm genome sequence is not publicly available, we used the published information from the NCBI database to design degenerate primers, using the online Primer3 tool (https://primer3.ut.ee, accessed on 10 May 2017) to amplify the partial gene sequences of V-ATPase A and beta-actin in wireworms (Table 1). The PCR primers for V-ATPase E and CHS1 were taken from published data [18,31].

The PCR mix consisted of genomic DNA, 1× PCR buffer (New England Biolabs, NEB, Whitby, ON, Canada), 1 μL of dNTP mix (10 mM each dNTP, NEB), 1 μL each of forward and reverse primers (5 μM) for each target gene, magnesium chloride (3 mM) and *Taq* DNA polymerase (1 U) (NEB) in a 50 μL reaction. The PCR conditions were as follows: initial denaturation at 94 °C for 5 min, cycle denaturation at 94 °C for 40 s, annealing at 51–62 °C for 40 s (Table 1), extension at 72 °C for 1 min for 40 cycles, and a final extension of 72 °C for 5 min. The PCR products were examined in 2% (*w*/*v*) agarose gel in 1 × Tris-acetate-EDTA buffer (10 mM Tris, 20 mM Acetate and 1 mM EDTA, pH = 8.0) and stained using GelRed^®^ (Biotium, Fremont, CA, USA). The individual amplicons were cloned into a pCR4-TOPO TA vector (Invitrogen by Life Technologies, Carlsbad, CA, USA) as per the manufacturer’s instructions, and positive clones of all the genes were sequenced using commercial sequencing services (TCAG Facilities, Toronto, ON, Canada). After the PCR products were confirmed by DNA sequencing, the purified PCR products were used as templates for *in vitro* dsRNA synthesis.

### 2.4. In Vitro dsRNA Synthesis

The PCR products were used as templates for the *in vitro* transcription of dsRNA using the designed primers (Table 1) of each target gene. Each primer contained a T7 polymerase promoter sequence (5′-TAATACGACTCACTATAGG-3′) at the 5′ end. The PCR products were analyzed on 2% (*w*/*v*) agarose gel, purified, and used as templates for *in vitro* transcription reactions. The *in vitro* transcription reactions (50 μL) were carried out using the MegaScript RNAi kit (Ambion Inc., Los Angeles, CA, USA), according to the manufacturer’s protocol. Briefly, the reaction was carried out at 37 °C for 2 h and nuclease digestion was undertaken using DNase I (2 U; Fermentas, Toronto, ON, Canada) and RNase with incubation at 37 °C for 1 h, followed by the purification of dsRNA using a column, according to the manufacturer’s protocol. The concentrations of dsRNA were estimated using NanoDrop 2000 Spectrophotometers (Thermo Fisher Scientific, Wilmington, DE, USA), and the samples were stored at −80 °C until further use. The dsRNA produced from the four target genes was ready for the feeding assay to evaluate gene silencing efficiency.

### 2.5. Delivery of dsRNA by Liquid Ingestion Method

**Figure 2:**
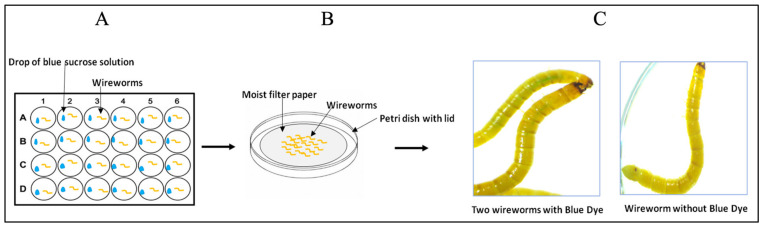
Stepwise liquid ingestion method. (**A**) Larvae feeding on 4 µL drop of blue-coloured sucrose solution in a 24-well culture plate. (**B**) Wireworms were incubated in a Petri dish containing moist filter paper after ingestion for microscopy observation. (**C**) Two larvae (left) show a blue abdomen and one larva (right) represents the control.

For the bioassay, the liquid ingestion method utilizes a 0.5% sucrose solution and liquid blue food colour dye (Food Colour, Club House). The dye assists in observing and tracking the intestinal tract colour change after the ingestion of dsRNA. Because the instar of the *Agriotes* larvae could not be identified visually other than size, all wireworms ranging from 6 mm to 18 mm were used (Figure 1C). Four biological replicates per target gene (V-ATPase A, V-ATPase E, CHS1, and beta-actin) were carried out, with 10 larvae per replicate, for a total of N = 40 wireworms per target gene. Wireworms were fed with the dsRNA of the target genes three times: on Day 1, Day 3, and Day 6. In this bioassay experiment, the three feeding times (Days 1, 3, and 6) were chosen to ensure sustained exposure to dsRNA while allowing for potential recovery periods. Two independent experiments were performed. The detailed procedure is described below and is illustrated in Figure 2.

a. Prior to feeding, wireworms were transferred to Petri dishes on moist filter paper and starved (without food) for 24 h in the dark at RT.
b. The blue-coloured solution was freshly prepared by adding 1 mL of 0.5% sucrose solution to nuclease-free water (Invitrogen) and 8 µL of blue food colour dye in a 1.5 mL microcentrifuge tube.
c. On Day 1, one wireworm larva was placed in each well of a 24-well culture plate. Then, 4 µL droplets containing 2 µL blue-coloured sucrose solution (prepared above) and 2 µL of dsRNA (1 µg) were added, ensuring contact with the larvae mouthparts (Figure 2A). For the negative control, a droplet containing 2 µL of nuclease-free water (instead of dsRNA) and 2 µL of blue-coloured sucrose solution was added. The plate was covered and incubated for 90 min in the dark at RT.
d. After the incubation, all the wireworms were transferred carefully with forceps from each well to a Petri dish (60 mm × 15 mm) (Figure 2B) and observed under a stereomicroscope (Leica, MZFLIII) to identify the ones that had ingested the blue-coloured sucrose solution (Figure 2C). The worms that showed a blue colour in their intestinal tract were only transferred to a new Petri dish (on moist filter paper), covered, and kept at RT in the dark for two days.
e. On Day 3, all the wireworms were transferred again from their Petri dishes to a new 24-well culture plate, and steps “c” and “d” were repeated for the second feeding. The filter paper at the bottom of the Petri dish was made moist by misting the filter paper lightly with nuclease-free water.
f. On Day 6, step “e” was repeated for the third feeding.

The entire experiment lasted for 30 days. Mortality, visibly misshapen phenotypes, and movement activity were recorded on Days 1, 6, 8, 12, 18, 20, 25, and 30 to assess the impact of exposure to the dsRNA treatment in wireworms over time. This design allowed us to observe the cumulative effects on the target organism’s development and behavior. To assess movement activity, each wireworm was independently observed for 1 min under a stereomicroscope at RT, and their real-time movement was recorded into four categories (more active; active; less active; and dead larvae) to estimate the effects of RNAi after the ingestion of dsRNA. The dead wireworms were counted and removed daily, and slices of carrot were placed after 15 days into the Petri dishes to provide additional nutrients.

All solutions must be prepared in nuclease-free water, and all tools used must be sterilized. It is crucial to keep the filter paper in the Petri dishes moist for the entire feeding experiment.

### 2.6. Gene Expression Analysis Using RT-qPCR

A quantitative reverse transcription polymerase chain reaction (RT-qPCR) experiment was conducted to further confirm the expressions of the most effective target gene (V-ATPase A) in wireworms. A total of 384 wireworms were used, and they were divided into two groups (fed dsRNA wireworms (treated) and non-fed dsRNA wireworms (negative control)), each containing 192 wireworms. The treated group was fed with 4 µL droplets containing 2 µL of blue-coloured sucrose solution (prepared above) and 2 µL of dsRNA (1 µg), while the negative control group were fed with 2 µL of nuclease-free water instead of dsRNA and 2 µL of blue-coloured sucrose solution, and they were fed for 5 days (Day 1 to Day 5). The aim of this continuous exposure to dsRNA for five days was to produce a steady-state dsRNA effect, ensuring a more reliable measurement of transcript levels. For the gene expression setup, a total of 280 live wireworms were collected on Day 1, Day 6, Day 10, Day 16, Day 20, and Day 25 after being fed the dsRNA of the V-ATPase A target gene (5 wireworms pooled as one sample; 4 biological replicates; 2 treatments; 6 time intervals). The collected samples were immediately frozen in liquid nitrogen and stored at −80 °C until further use. Additionally, the number of dead wireworms that ingested the dsRNA were counted at 6 time intervals; however, due to uncertainty regarding the RNA integrity of dead wireworms, these were not included in the gene expression analysis.

Total RNA from the live fed dsRNA and non-fed dsRNA wireworms was isolated using the RNAeasy Mini kit (Qiagen Inc., Germantown, MD, USA) according to the manufacturer’s instructions. RNA concentration and integrity were analyzed using a NanoDrop 2000 Spectrophotometer (Thermo Fisher Scientific, DE, USA) and 1% agarose gel, respectively. The First-Strand cDNA was synthesized from 1 µg total RNA using SuperScript III Reverse Transcriptase (Invitrogen) and stored at −80 °C until use in subsequent gene expression analyses.

RT-qPCR was performed using SYBR green and gene-specific primers (Table 1) that were designed using the online Primer3 tool (https://primer3.ut.ee, accessed on 29 August 2018). The RT-qPCR primers used and effective siRNA sites are marked in Figure S1. The siRNA sites of the dsRNA of the V-ATPase A target gene were predicted by the online siRNA Wizard software 3.1 (https://www.invivogen.com/sirnawizard/design.php, accessed on 20 November 2024). The linear ranges and amplification efficiencies were determined across five 10-fold serial dilutions of cDNA to generate a standard curve using the gene-specific primers, and correlation coefficients were analyzed. In this study, the beta-actin gene was used as an internal control gene to normalize and evaluate relative gene expression. The beta-actin gene was chosen as the reference gene because it showed the most stable expression among the samples under different serial dilutions. Each qPCR sample contained 2 µL of the synthesized cDNA, 1 µL of each primer (forward and reverse, 5 µM), 6 µL of nuclease-free water, and 10 µL of iTaq Universal SYBR Green Supermix (2X, Bio-Rad Laboratories, Hercules, CA, USA). RT-qPCR was conducted using a StepOnePlus™ Real-Time PCR System (Applied Biosystem by Life Technologies, Carlsbad, CA, USA). All the reactions were performed under the following RT-qPCR thermal cycling conditions: 1 min at 95 °C, 30 s at 95 °C for denaturation, followed by 30 s at 65 °C for annealing (40 cycles), and finally the generation of a melting curve consisting of a single peak to rule out non-specific products and primer–dimer formations. The mean of the Ct values for 3 technical replicates was calculated and the fold change in the expression of the target gene was calculated using the 2^−∆∆Ct^ method [47]. Three independent experiments were performed. For statistical analysis, the data are expressed as mean ± standard deviation (SD). A one-way analysis of variance (ANOVA) was performed to identify the significance of the differences between the groups using Tukey’s Honest Significant Difference (HSD) test (*p* < 0.05) and Minitab software Version 22.

## 3. Results

### 3.1. Wireworm Rearing

It is a challenging task to collect samples when studying wireworms because they may burrow as deep as 24 inches into the soil, and they migrate upward and downward in the soil depending on soil moisture and temperature throughout the season [48]. Therefore, effectively maintaining wireworms for experimentation year-round is essential. In this study, approximately 4000 wireworms were collected from fields and reared continuously in a laboratory for two years. Morphologically, wireworms are yellow and/or light brown with hard, shiny skin (Figure 1A). There are three sets of legs near the head and two small dark spots on the upper surface of the last segment of the body (Figure 1A), which are distinguished characteristics of *Agriotes* species [1,49,50]. This study established the feasible laboratory conditions for maintaining wireworms collected from fields. Wireworms, including all instars (6–18 mm), were kept in containers with moist soil and food (Figure 1B,C) both in a dark growth chamber at RT for at least six months, as well as at a lower temperature (6–8 °C) in the fridge for long-term storage (up to two years). Wireworms were observed once a week to evaluate survival rate, and food was replaced as needed throughout the study. It is important to keep the soil moist throughout the period.

### 3.2. Characterization of Agriotes spp. in Eastern Canada

The morphology of the three species of *Agriotes*—*A. obscurus*, *A. lineatus*, and *A. sputator*—is very similar and difficult to distinguish visually. To distinguish these three *Agriotes* species, a partial mtCOI gene was amplified using PCR with the universal invertebrate primers LCO1490 and HCO2198 and sequenced. The DNA sequences were subjected to NCBI BLAST database, revealing that all the collected samples from the P.E.I. and Truro regions were *A. sputator* species with 98% identity, where 89% identity with *A. lineatus* and 88% with *A. obscurus* species (Figure S2). Furthermore, the multiple sequence alignment using ClustalW (BioEdit) with the reference sequence of the mtCOI gene (NCBI accession number: KR124576.1) revealed that all our samples were homologous with *A. sputator* (sequence 1), as compared with *A. lineatus* or *A. obscurus* (Figure S2).

### 3.3. Cloning and Sequencing of the Target Genes

Four target genes, V-ATPase A, V-ATPase E, CHS1, and beta-actin, were used in the present study (Table 1). The partial gene sequences were successfully PCR-amplified using degenerate or published primers and cloned into a plasmid vector (pCR4-TOPO TA vector, Invitrogen). They were subsequently sequenced and subjected to NCBI BLASTX database. Since genomic information for *Agriotes* spp. is unavailable, the sequences of all four target genes with the highest similarity and maximum identity (%) to those of other insect species were used as represented in Table 2. All partial sequences generated for each target gene in this study were submitted to GenBank (NCBI) (accession number OR365161 for V-ATPase A; accession number OR365162 for V-ATPase E; accession number OR365163 for beta-actin; and accession number OR365164 for CHS1) and will become available once the research is published.

Additionally, our laboratory has generated *de novo* transcriptome assemblies from the different developmental stages of *A. sputator* species (larva and adult (female and male) click beetle stages) to identify differentially expressed genes and their functional pathways [51]. The complete coding sequences of all four target genes from our in-house RNA-Seq data are being processed; we will soon submit the full-length sequences of these target genes to Genbank.

### 3.4. Evaluation of the Liquid Ingestion Method

Since no report on the delivery of dsRNA to wireworms is available, we developed a liquid ingestion method to evaluate its efficiency and assess the effect of dsRNA on the wireworm larvae. Using this method, 4 µL droplets containing 2 µL of the dsRNA of the genes of interest (1 µg) and 2 µL of blue-coloured sucrose solution were administered, ensuring contacted with the mouthparts of the larvae (Figure 2A,B). Our feeding experiments consistently showed a high percentage of wireworms ingesting the blue-coloured sucrose solution, close to 100%. The wireworms presented a blue coloration in their abdomen (Figure 2C), indicating that the dsRNA had also been ingested and passed through the gut. During feeding, we found that some of the smallest larvae (less than 8 mm long) died after Day 1 or Day 2 due to the handling procedures, as they were tiny and delicate. Therefore, larvae less than 8 mm are not recommended for this liquid ingestion method. Our experience showed that wireworm sizes larger than 8 mm are suitable for this liquid ingestion method and can successfully acquire dsRNA orally.

### 3.5. Effect of dsRNA in Wireworms

**Figure 3:**
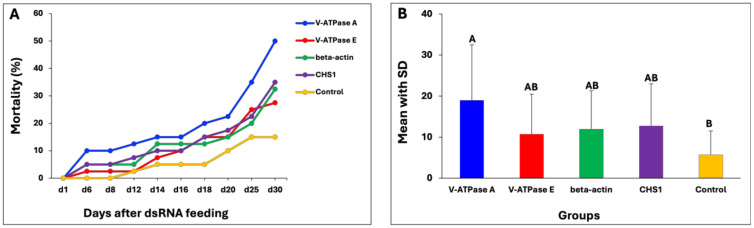
dsRNA efficiency of four target genes in wireworms. (**A**) The line graph shows larval mortality (%) after ingesting dsRNA of four target genes compared with the negative control, recorded on different days. (**B**) The bar chart plot shows the mean with SD of mortality (%), generated by combining all time intervals. Statistical comparison was undertaken using Tukey’s HSD test. Different letters represent significant differences at *p*-value < 0.05.

In the bioassay, the dsRNA of the four target genes was administered to the wireworms three times, on Days 1, 3, and 6, using the liquid ingestion method described above to investigate the functionality of RNAi, while the negative control wireworms ingested a blue-coloured sucrose solution with nuclease-free water. The phenotypic effects recorded included larval mortality, changes in movement activity, and visibly misshapen phenotypes over 30 days. For all target genes, wireworms after being fed dsRNA started to die on the sixth day, and significant mortalities were observed by Day 16 (Figure 3A). The dsRNA products of V-ATPase A, V-ATPase E, beta-actin, and CHS1 fed to the wireworms resulted in mortality; however, the mortality rate and the timing of death varied. The dsRNA of the V-ATPase A gene caused over 50% mortality after 30 days of feeding, while the dsRNAs of V-ATPase subunit E, beta-actin, and CHS1 resulted in approximately 28%, 33%, and 35% mortality, respectively. The mortality between the V-ATPase A group and the negative control group was found to be statistically significant (*p* = 0.014) (Figure 3B). However, no significant differences were found between the dsRNAs of the other three target genes of V-ATPase E (*p* = 0.179), beta-actin (*p* = 0.089), and CHS1 (*p* = 0.076), or the negative control group. Only 15% mortality after the 30-day ingestion experiment was observed in the negative control group.

**Figure 4:**
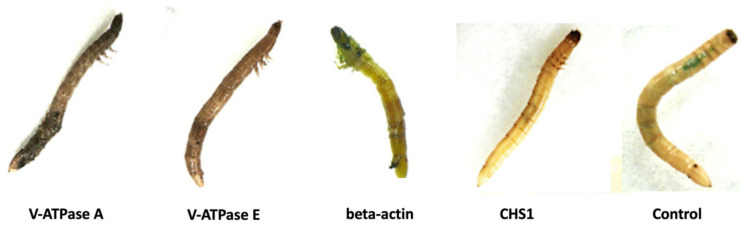
Malformation of wireworms after ingestion of dsRNA of the four target genes compared with the control.

As the mortality triggered by dsRNA was observed from the sixth day for all target genes, the movement activity of the larvae was also recorded on Day 1, Day 6, Day 12, Day 18, Day 25, and Day 30 (Figure S3). Their real-time movement activities were divided into four categories (more active; active; less active; and dead larvae). Starting from Day 6, the movement activity of larvae was a bit reduced as a result of feeding on the dsRNA of all four target genes, compared with the movement activity of the negative control larvae. On Day 18, the wireworms fed V-ATPase A, V-ATPase E, beta-actin, and CHS1 dsRNA showed a substantial inhibition of functions and became less active, and some larvae were found dead, while the negative control larvae were found to be equally active throughout the feeding experiment, except for two and three wireworms found dead on Day 25 and Day 30, respectively. In addition, some wireworms showed malformed phenotypes after being fed with the dsRNA of the target genes. For the first 6–8 days, the physical appearance of the larvae was normal for all four target genes. However, deformed phenotypes, such as shrunken, melanized, and hyperpigmented cuticle abnormalities, were observed in wireworm larvae that were fed with the dsRNA of the V-ATPase A, V-ATPase E, and beta-actin target genes, while no deformed phenotypes were observed in the control group (Figure 4).

### 3.6. dsRNA Targeting V-ATPase A in Wireworms

**Figure 5:**
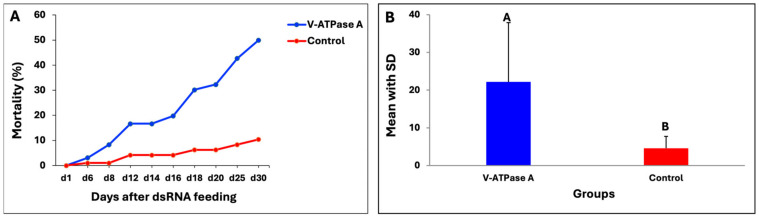
RNAi efficiency of dsRNA of V-ATPase A in wireworms. (**A**) The line graph shows larval mortality (%) after ingesting the dsRNA of the V-ATPase A gene compared with the controls, recorded on different days. (**B**) The bar chart plot shows the mean with SD of mortality (%), generated by combining all time intervals. Statistical comparison was calculated using Tukey’s HSD test. Different letters represent significant differences at *p* < 0.05.

Since the dsRNA of the V-ATPase A gene showed the highest mortality and reduced the movement activity of living wireworms (Figure 3 and Figure S3), further assessments were carried out to confirm the gene inhibition effect of the dsRNA targeting V-ATPase A. To undertake this, a total of 96 wireworms were selected (24 wireworms per experiment, with a total of 4 independent experiments) and fed with the dsRNA of V-ATPase A in this bioassay experiment. Again, the dsRNA of V-ATPase A caused more than 50% mortality after the 30-day ingestion experiment (Figure 5A) and resulted in a significant lethal effect (*p* = 0.004) on dsRNA-fed wireworms compared with the control group (Figure 5B).

### 3.7. Gene Expression of V-ATPase A Target

**Figure 6:**
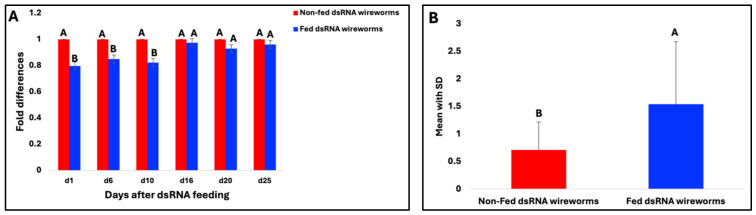
(**A**) Comparison of V-ATPase A gene expression level between treated (fed dsRNA) and negative control (not-fed dsRNA) wireworms. Samples were collected on Day 1, Day 6, Day 10, Day 16, Day 20, and Day 25 after feeding started. Each bar represents the fold differences of the V-ATPase A target gene in the treated and negative control wireworms, which were calculated using the 2^−ΔΔCT^ method. (**B**) The bar chart plot shows the mean with SD of the number of dead wireworms, generated by combining all time intervals that were found in the treated and negative control groups. Pairwise statistical comparison was calculated using Tukey’s HSD test. Different letters represent significant differences at *p* < 0.05.

RT-qPCR was performed to ascertain if the mortality of the dsRNA of the V-ATPase A-treated wireworms correlated with the down-regulation of the corresponding transcripts. Since the wireworms were fed with the dsRNA of the V-ATPase A target gene for five days continuously, the live wireworm samples were collected on Day 1 (one day after ingestion), Day 6, Day 10, Day 16, Day 20, and Day 25. The ingestion of the dsRNA of V-ATPase A resulted in significant reductions in corresponding transcript levels compared to the control group on Day 1, Day 6, and Day 10 (Figure 6A). The results confirmed that the ingested dsRNA resulted in RNAi, efficiently inhibiting V-ATPase A gene activity in wireworms. However, no significant reduction in transcript abundance was observed on Day 16, Day 20, or Day 25 between the fed and non-fed dsRNA wireworms. Since the wireworms that died after feeding on the dsRNA of the V-ATPase A gene were not included in the gene expression analysis (because of the uncertainty of the RNA integrity of the dead samples), the number of dead wireworms was also counted throughout the feeding experiment. The pairwise statistical comparison was significant (*p* = 0.04) between the fed and non-fed dsRNA wireworms using Tukey’s HSD test (Figure 6B). This result indicates that the dsRNA of the V-ATPase A gene triggered the RNAi effects in wireworms.

## 4. Discussion

Since *Agriotes* species have long life cycles, observing their response to changes in temperature, soil moisture, and other environmental factors is a laborious task, as these factors modify their habitat and influence the duration of their life cycles. Since wireworms cannot be propagated in laboratory conditions easily, collecting them from fields and properly rearing them in the laboratory is a prerequisite. The present study offers a detailed protocol for the rearing of wireworms in the laboratory. It is simple, easy to handle, and offers a high survival rate for up to a year.

The development from wireworms to click beetles takes 4–5 years [52,53], though the life cycle duration varies among species. Additionally distinguishing larvae of closely related *Agriotes* species based on morphological characteristics is another challenge. In this study, we used a molecular approach using the PCR method to distinguish the field-collected *Agriotes* species from Eastern Canada. Therefore, we amplified the partial mtCOI genes using the universal invertebrate primers LCO1490 and HCO2198 and sequenced them. The results revealed that all sequenced samples were homologous with *A. sputator* species, suggesting that *A. sputator* is predominant and widely distributed in the region of Eastern Canada. Vernon and van Herk [1] also reported that *A. sputator* is the most common pest found in fields of potatoes in Eastern Canada, based on pheromone-based traps. Sequencing the PCR products amplified with the universal mtCOI primers [43] facilitates the reliable identification of *Agriotes* species via comparing the generated sequences with those deposited in GenBank. Staudacher et al. also reported using the mtCOI gene to differentiate nine *Agriotes* species found in Central Europe [7].

The limits of commercial pesticides have increased the demand for innovative and environmentally friendly plant protection strategies. In recent years, RNAi triggered by dsRNA has evolved as a promising strategy for controlling insects in a species-specific manner, and has shown potential for pest management [54,55,56,57]. However, efficient and successful applications of RNAi are challenging in some insects. The first challenge is to identify the potential target genes. For instance, Baum et al. [18] screened 290 potential target genes in the Western corn rootworm *Diabrotica virgifera virgifera*, analysing the ingestion of an artificial diet with dsRNAs. The study reported that only 14 genes demonstrated precise down-regulation, which led to remarkable larval stunting and mortality. Since the wireworm genome of *Arigotes* spp. is unavailable, finding target genes in wireworm for RNAi is challenging. Secondly, the dsRNA delivery method is critical for studying gene silencing functions. To date, several approaches have been used to deliver dsRNA molecules to insects, all of which have suitable applications and limitations. The most popular method for dsRNA delivery is microinjection into the insect’s hemocoel [58,59,60,61]. Nevertheless, the high cost of the microinjection system is prohibitive for most laboratories. It is also labor-intensive and not suitable for high-throughput applications. Delivering dsRNA orally by feeding has also been reported in many insect taxa [39]. The benefits of feeding methods include reduced stress for organisms and ease of use for small insects. The successful oral delivery of dsRNA into insects via feeding has been achieved for many insects: the light brown apple moth (*Epiphyas postvittana*) [62], the diamondback moth (*Plutella xylostella*) larvae [63], the emerald ash borer (*Agrilus planipennis*) [64], and the honey bee [65,66]. Alternatively, soaking has also been used to deliver dsRNA into *Caenorhabditis elegans* nematodes [67] and insect cell lines [68,69,70]. The soaking approach has numerous advantages over injection, such as no specialized equipment being required and more significant numbers of worms being treated in each experiment. However, the soaking method is more expensive than injection or feeding, owing to the large volume of dsRNA used for each experiment. RNAi can also be induced by feeding insects with transgenic plants expressing dsRNA [18,33]. Transgenic plants, however, are time-consuming to produce and not easy to release due to biosafety-related regulations in different countries [22].

In this study, we offer a successful liquid ingestion method for the delivery of dsRNA into wireworms that allowed us to screen potential target genes. This liquid ingestion method is the most efficient since it only requires a small volume of the test solution, and many wireworms can be handled simultaneously. Moreover, the necessary experimental setup is quick and straightforward, and the presence of a visible blue dye in the ingestion solution allows individual wireworms to be readily distinguished. This advantage is significant because knowing which wireworms have ingested the dsRNA will ensure the accuracy of the RNAi results. It can also be calibrated to quantify the susceptibility of other species of Coleoptera to dsRNA or other insecticidal compounds.

Furthermore, we investigated four target genes (V-ATPase subunit A, V-ATPase subunit E, CHS1, and beta-actin) that are important for growth and development in wireworms. These genes were chosen based on prior findings indicating that the silencing of these target genes by RNAi could lead to insect death [18,31,46]. The *in vitro* synthesised dsRNA of target genes was administered using our developed liquid ingestion method. It successfully triggered the silencing of target genes and caused considerable death in treated wireworms, indicating an effective RNAi response in wireworms as a result of feeding. The data presented here show that RNAi against V-ATPase subunit A with 1 µg dsRNA concentration caused up to 50% mortality in wireworm larvae in two independent experiments, compared to the negative control (Figure 3 and Figure 5). Additionally, wireworms showed malformed phenotypes (Figure 4), confirming the target gene’s involvement in specific physiological processes. However, the mortality rates were not found to be significant when using dsRNA of V-ATPase subunit E, beta-actin, and CHS1, with rates of 28%, 33%, and 35%, respectively (Figure 3). These results suggest that the lack of significant RNAi silencing utilizing V-ATPase E, CHS1, and beta-actin target genes may be due to a lower dsRNA dose used in the wireworms. Previous studies have shown that in order to trigger an RNAi response in a variety of insects, the ideal dsRNA concentration or dosage is necessary [36,71,72]. It is very challenging to accurately evaluate the amount of dsRNA ingested by wireworms because certain factors can affect this, such as low or variable dosages ingested by individual insects, feeding frequency, and size, which may all limit the effectiveness of RNAi in midgut transcripts. Additionally, the gastrointestinal tract system and physiology can also influence the actual RNAi dose that reaches the midgut epithelium [39]. The gene expression level of the V-ATPase A target gene was also confirmed using RT-qPCR, which showed a significant decrease in the relative expression level of V-ATPase A mRNA on Days 1, 6, and 10 compared with the expression in non-fed dsRNA wireworms (Figure 6A). Additionally, the number of wireworms that died after being fed dsRNA was also counted until Day 25 and found to be significant (Figure 6B). However, no significant down-regulation of the V-ATPase A gene at the mRNA level was observed on Days 16, 20, or 25 (Figure 6A). It is noteworthy that the feeding setups between the bioassay and gene expression experiments were different, as detailed in the Materials and Methods. In the bioassay experiment, wireworms were fed with dsRNA three times (on Days 1, 3, and 6) to evaluate the phenotypic effects of dsRNA treatment over time. The findings showed that the dsRNA targeting the V-ATPase A gene caused up to 50% mortality in wireworms (Figure 3 and Figure 5). In contrast, in the gene expression experiment, wireworms were continuously exposed to dsRNA for five consecutive days (Days 1 to 5) to establish a steady-state dsRNA effect. Under this setup, the dsRNA targeting the V-ATPase A gene resulted in significant gene expression inhibition on Days 1, 6, and 10, alongside a substantial number of dead wireworms (Figure 6). Also, we conducted this RNAi experiment with a blue-coloured sucrose solution with nuclease-free water as a control. Other reports have used dsGFP (dsRNA targeting green fluorescent protein gene) as a control [33,34,36,64], which could be adapted in future investigations. In recent years, it has been demonstrated that Coleopteran insects, such as the Western corn rootworm (*Diabrotica virgifera virgifera*), the Colorado potato beetle (*Leptinotarsa decemlineata*), and red flour beetle (*Tribolium castaneum*), are quite susceptible to RNAi and can be successfully killed by being fed lethal dsRNA targeting specific genes. For instance, Baum et al. found that dsRNA targeting V-ATPase A and V-ATPase E caused significant larval mortality in Western corn rootworm and Colorado potato beetle bioassays [18]. Interestingly, it has been illustrated that the effectiveness of RNAi-mediated pest control varies greatly among different groups of insects [37,72,73]. Several factors impacting the efficacy of RNAi in insects have been found, such as the role of the gut microbiota, the stability of dsRNA in the digestive tract, the effectiveness of the cellular uptake of dsRNA, endosomal trapping, and the activity of the core RNAi machinery [74,75,76,77]. Therefore, a comprehensive understanding of the molecular mechanisms affecting RNAi efficiency in wireworms is still lacking.

This is our first attempt to demonstrate an efficient feeding methodology to allow the screening and selection of the most efficient target genes that cause larval mortality. Recently, our lab generated *de novo* transcriptome profiles of wireworms and adult click beetles of *Agriotes sputator* using Illumina paired-end technology [51], and many genes involved in metabolic processes, growth development, and reproduction were identified during different stages of insect life. Future studies can focus on the molecular analysis of the wireworm; more potential target genes can be screened from *de novo* transcriptomes, and gene expression levels with different concentrations of dsRNA or combinations of one or more RNAi target genes will be assessed using RT-qPCR to better understand these findings.

RNAi is a promising approach that aligns well with the growing interest in sustainable and environmentally friendly pest management strategies because of its species-specific action and natural degradation. Nevertheless, the commercialization of insecticidal dsRNA for the control of insect pests is still challenging. Only one product has been approved in Canada (2016) and the United States of America (2017) so far: the transgenic GM-maize SmartStax PRO, which expresses dsRNA targeting the *Dv-snf7* gene in the Western corn rootworm [23]. The RNAi-based strategy, however, faces several hurdles, including expensive capital requirements, the public acceptance of GM crops, and technical difficulties in changing some crop species. Due to these difficulties, researchers are looking into alternative methods, which mostly use exogenous dsRNA against target pests. However, the development of low-cost methods for the large-scale production of dsRNA is necessary for crop protection. Data show that 2–10 g of dsRNA per hectare will be required for effective crop protection [78]. Recently, large-scale dsRNA production has been made possible via *in vivo* production systems using engineered microbes and *in vitro* synthesis techniques using RNA polymerase [79]. There are currently three methods for applying dsRNA in the field that show a lot of promise: trunk injection, root irrigation, and foliar spraying [79]. However, it is unclear which method is effective for controlling pests, as the exogenously applied dsRNA would be exposed to the environment; hence, more research is needed. This study demonstrated that many target genes could be screened using our developed liquid ingestion method in the laboratory setting in future, and this method could also be used to examine some potential RNAi mechanisms in wireworms. Thus, future studies should focus on the molecular analysis of the wireworm, offer a framework for developing a new generation of species-specific and environmentally friendly pest control strategies.

## 5. Conclusions

We have reported the establishment of a liquid ingestion method used to deliver dsRNA in wireworms of *Agriotes* species. The method is simple, fast, and cost-effective, and can be used on a large scale. It was demonstrated that *in vitro* synthesized dsRNA can be ingested by wireworms using this feeding method, which could trigger gene silencing and subsequently affect wireworm development. We also identified four target genes that can be used as RNAi targets for wireworm control. Our findings suggest that V-ATPase subunit A could alone be a promising target gene for the suppression of wireworm populations.

## Figures and Tables

**Table 1:** PCR primers were used to amplify the four target genes and their dsRNA production for the feeding experiment. The underlined and bold font shows the T7 promoter sequence.

Gene Name	PCR Primer Sequences (5′–3′)	Annealing Tm (°C)	Product Length
	PCR amplification and cloning		
V-ATPase subunit A	F: RCGNGGNAAYGARATGTCNG R: GCCATCATNGANACRTTRTA	57	207 nt Own design
V-ATPase subunit E	F: ATCATGGARTACTAYGARAARAARGAG R: GTTGCGWCCGAASAGMGCVTTWCGGATCTSSGG	55	501 nt [18]
CHS1	F: TTYGARTAYGCNATHGGNCAYTGG R: CCANCKRTCYTCNCCYTGRTCRTAYTG	62	192 nt [31]
beta-actin	F: TCNATHATGAARTGYGAYGT R: CNCCDATCCANACNGARTAY	51	187 nt Own design
	*In vitro* dsRNA synthesis		
V-ATPase subunit A	F: **TAATACGACTCACTATAGG**RCGNGGNAAYGARATGTCNG R: **TAATACGACTCACTATAGG**GCCATCATNGANACRTTRTA		
V-ATPase subunit E	F: **TAATACGACTCACTATAGG**ATCATGGARTACTAYGARAARAARGAG R: **TAATACGACTCACTATAGG**GTTGCGWCCGAASAGMGCVTTWCGGATCTSSGG		
CHS1	F: **TAATACGACTCACTATAGG**TTYGARTAYGCNATHGGNCAYTGG R: **TAATACGACTCACTATAGG**CCANCKRTCYTCNCCYTGRTCRTAYTG		
beta-actin	F: **TAATACGACTCACTATAGG**TCNATHATGAARTGYGAYGT R: **TAATACGACTCACTATAGG**CNCCDATCCANACNGARTA		
	RT-qPCR		
V-ATPase subunit A	F: CGAGCTCTCGGTGGAAATC R: AAATGGAAGCTTCACGAGCA	65	107 nt Own design
beta-actin	F: CGCCAACACTGTACTCTCTGG R: CGATGATCTTGATCTTGATGG	65	110 nt Own design

**Table 2:** The top three BLASTX hits (NCBI database) based on the DNA sequences of the amplified PCR fragments of the four target genes.

Target Genes	BLASTX Hits (Identity in %) with Reported Species
V-ATPase subunit A	97%: *Tribolium castenum* (XP_976188.1) 97%: *Diabrotica virgifera virgifera* (XP_050506367.1) 95%: *Dendroctonus ponderosae* (XP_048525241.1)
V-ATPase subunit E	79%: *Agrilus planipennis* (XP_018320654.1) 78%: *Photinus pyralis* (XP_031347368.1) 70%: *Tribolium castenum* (XP_970621.1)
CHS1	96%: *Helicoverpa zea* (AAG09738.1) 96%: *Anabrus simple* (WED299771) 95%: *Phenacoccus solenopsis* (AIE17035.1)
beta-actin	100%: *Agasicles hygrophila* (ALP48321.1) 100%: *Maruca vitrata* (QYY49471.1) 100%: *Rhipicephalus microplus* (AAS09968.1)

## Data Availability

The partial nucleotide sequences of the RNAi target genes have been deposited at NCBI, as indicated in the Materials and Methods section. The datasets generated or analysed during this study are available from the corresponding author upon reasonable request.

## Supplementary Materials

The supporting files can be downloaded at: https://www.mdpi.com/article/10.3390/insects15120983/s1, Figure S1: Schematic representation of the dsRNA fragment of V-ATPase A target gene, showing siRNA target sites and RT-qPCR primer pair used to quantify transcript mRNA level; Figure S2: Multiple sequence alignment using ClustalW, created using the partial DNA sequences of the mtCOI gene PCR products from wireworms collected from fields in Eastern Canada; Figure S3: Effects of dsRNA on the movement activity of wireworm larvae using four target genes and control wireworms.
