# Supplementary material for: Gene Silencing via Ingestion of Double-Stranded RNA in Wireworm of Agriotes Species
Source: Insects. 2024 Dec 11;15(12):983. doi: 10.3390/insects15120983 (PMC11679789; doi:10.3390/insects15120983)
Supplement: Supplementary file 1 [file insects-15-00983-s001.zip › Figure S3.pdf]

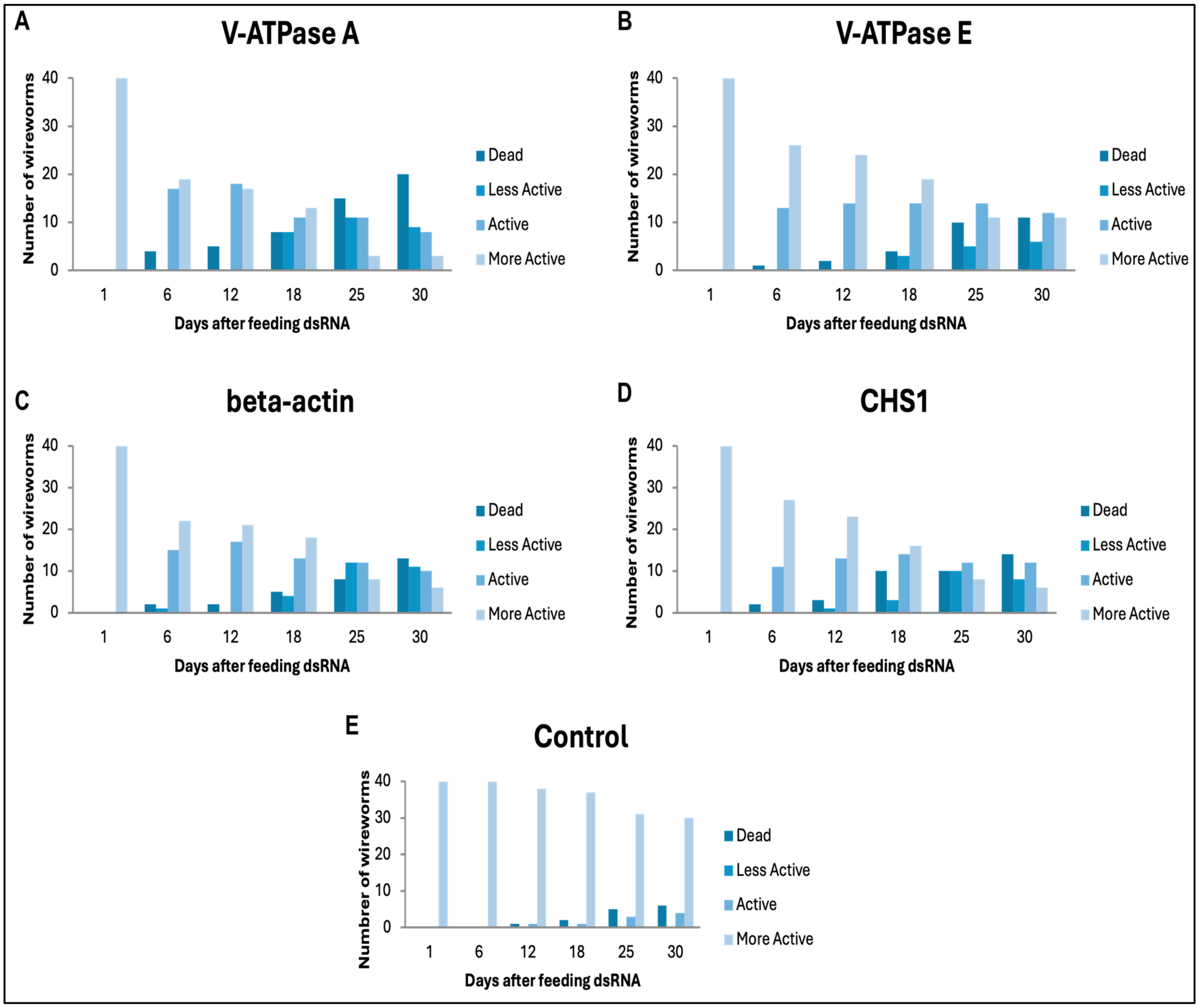

Figure S3. Effects of dsRNA on the movement activity of wireworm larvae using four target genes and control wireworms. X-axis: movement activity of wireworm on different days after feeding dsRNA; Y-axis: number of wireworms in four different categories (dead, less active, active and more active). A total of 40 wireworms were used per target gene (10 wireworms per replicate).
