# Supplementary material for: Gene Silencing via Ingestion of Double-Stranded RNA in Wireworm of Agriotes Species
Source: Insects. 2024 Dec 11;15(12):983. doi: 10.3390/insects15120983 (PMC11679789; doi:10.3390/insects15120983)
Supplement: Supplementary file 1 [file insects-15-00983-s001.zip › Figure S1.pdf]

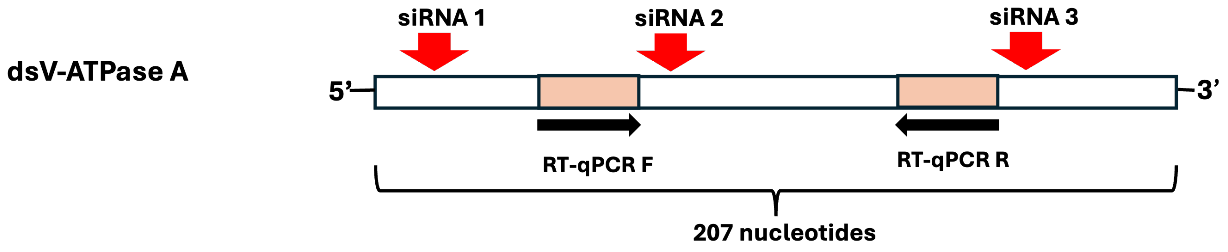

Figure S1. Schematic representation of the dsRNA fragment of V-ATPase A target gene, showing siRNA target sites and RT-qPCR primer pair used to quantify transcript mRNA level. Red arrows indicate siRNA target sites, and RT-qPCR F and RT-qPCR R are primers represented by black arrows.
