# Supplementary material for: Gene Silencing via Ingestion of Double-Stranded RNA in Wireworm of Agriotes Species
Source: Insects. 2024 Dec 11;15(12):983. doi: 10.3390/insects15120983 (PMC11679789; doi:10.3390/insects15120983)
Supplement: Supplementary file 1 [file insects-15-00983-s001.zip › Figure S2.pdf]

```
                                         10        20        30        40        50        60        70        80        90       100
                              ....|....|....|....|....|....|....|....|....|....|....|....|....|....|....|....|....|....|....|....|
Agriotes_sputator_KR124576.1  ATGCTAGGAACATCTCTAAGATTACTAATTCGTGCTGAATTAGGTAACCCTGGGTCATTAATCGGAAATGACCAGATTTATAACGTTATTGTAACAGCAC
Agriotes_lineatus_KJ966025.1  ..............AT.....................................A...C......................C...................
Agriotes_obscurus_KM442197.1  ..............A......................................A...C...................C..C..T.....C..........
PEI_1                         ....................................................................................................
PEI_2                         ....................................................................................................
PEI_3                         ....................................................................................................
PEI_4                         ....................................................................................................
PEI_5                         ....................................................................................................
PEI_6                         ....................................................................................................
PEI_7                         ....................................................................................................
PEI_8                         ....................................................................................................
PEI_9                         ....................................................................................................
Truro_1                       ....................................................................................................
Truro_2                       ....................................................................................................
Truro_3                       ....................................................................................................
Truro_4                       ....................................................................................................
Truro_5                       ....................................................................................................
Truro_6                       ....................................................................................................

                                        110       120       130       140       150       160       170       180       190       200
                              ....|....|....|....|....|....|....|....|....|....|....|....|....|....|....|....|....|....|....|....|
Agriotes_sputator_KR124576.1  ATGCTTTCATTATAATTTTCTTCATAGTAATACCAATTATAATTGGGGGATTTGGAAATTGATTAGTCCCTTTAATGCTTGGTGCCCCAGATATAGCCTT
Agriotes_lineatus_KJ966025.1  ..........C...........T...........T..C....................C...........A......T.A.................A..
Agriotes_obscurus_KM442197.1  ......................T...........T..C....................C...........A.....AT.A.................A..
PEI_1                         ....................................................................................................
PEI_2                         ....................................................................................................
PEI_3                         ....................................................................................................
PEI_4                         ....................................................................................................
PEI_5                         ....................................................................................................
PEI_6                         ....................................................................................................
PEI_7                         ....................................................................................................
PEI_8                         ....................................................................................................
PEI_9                         ....................................................................................................
Truro_1                       ....................................................................................................
Truro_2                       ....................................................................................................
Truro_3                       ....................................................................................................
Truro_4                       ....................................................................................................
Truro_5                       ....................................................................................................
Truro_6                       ....................................................................................................

                                        210       220       230       240       250       260       270       280       290       300
                              ....|....|....|....|....|....|....|....|....|....|....|....|....|....|....|....|....|....|....|....|
Agriotes_sputator_KR124576.1  TCCCCGAATAAACAATATGAGATTTTGATTTCTTCCACCTTCATTGTCTCTTCTATTAATGAGAAGAATTGTTGAAAATGGTGCAGGAACAGGATGAACA
Agriotes_lineatus_KJ966025.1  C..A..............A....C.....CT.A..C.....CC.C..C..G..T.....A......................................T
Agriotes_obscurus_KM442197.1  C..A..............A....C..G...T.A..T..A..CC.C..C..A..T.....A......................................T
PEI_1                         ....................................................................................................
PEI_2                         ....................................................................................................
PEI_3                         ....................................................................................................
```

```
PEI_4                         ....................................................................................................
PEI_5                         ....................................................................................................
PEI_6                         ....................................................................................................
PEI_7                         ....................................................................................................
PEI_8                         ....................................................................................................
PEI_9                         ....................................................................................................
Truro_1                       ....................................................................................................
Truro_2                       ....................................................................................................
Truro_3                       ....................................................................................................
Truro_4                       ....................................................................................................
Truro_5                       ....................................................................................................
Truro_6                       ....................................................................................................

                                       310       320       330       340       350       360       370       380       390       400
                              ....|....|....|....|....|....|....|....|....|....|....|....|....|....|....|....|....|....|....|....|
Agriotes_sputator_KR124576.1  GTGTACCCCCCTCTTTCATCTAATATTGCCCATAGAGGATCCTCAGTAGATCTAGCAATTTTCAGACTTCACTTAGCAGGTATTTCCTCTATTCTAGGGG
Agriotes_lineatus_KJ966025.1  ...........A........A....................A........................T.A...........A..C..A..A........A.
Agriotes_obscurus_KM442197.1  ...........A........A....................A.....T..................T.A...........A..C..A..A........A.
PEI_1                         ....................................................................................................
PEI_2                         ....................................................................................................
PEI_3                         ....................................................................................................
PEI_4                         ....................................................................................................
PEI_5                         ....................................................................................................
PEI_6                         ....................................................................................................
PEI_7                         ....................................................................................................
PEI_8                         ....................................................................................................
PEI_9                         ....................................................................................................
Truro_1                       ....................................................................................................
Truro_2                       ....................................................................................................
Truro_3                       ....................................................................................................
Truro_4                       ....................................................................................................
Truro_5                       ....................................................................................................
Truro_6                       ....................................................................................................

                                       410       420       430       440       450       460       470       480       490       500
                              ....|....|....|....|....|....|....|....|....|....|....|....|....|....|....|....|....|....|....|....|
Agriotes_sputator_KR124576.1  CAGTAAATTTTATTTCAACAGTTATTAATATACGATCAACAGGAATCACATTTGATCGAATACCTTTATTTGTATGAGCAGTAGCAATCACAGCTTTATT
Agriotes_lineatus_KJ966025.1  ......................A.......................T.......................C..T..............T..T..AC....
Agriotes_obscurus_KM442197.1  ......................A.......................T..........................T........G........T..AC..C.
PEI_1                         ....................................................................................................
PEI_2                         ....................................................................................................
PEI_3                         ....................................................................................................
PEI_4                         ....................................................................................................
PEI_5                         ....................................................................................................
PEI_6                         ....................................................................................................
PEI_7                         ....................................................................................................
PEI_8                         ....................................................................................................
PEI_9                         ....................................................................................................
Truro_1                       ....................................................................................................
Truro_2                       ....................................................................................................
Truro_3                       ....................................................................................................
```

```
Truro_4                          ....................................................................................................
Truro_5                          ....................................................................................................
Truro_6                          ....................................................................................................

                                         510       520       530       540       550       560
                                 ....|....|....|....|....|....|....|....|....|....|....|....|....|
Agriotes_sputator_KR124576.1     GCTACTTTTATCTCTACCAGTATTAGCTGGGGCAATTACTATGTTACTAACAGACCGTAATCTAA
Agriotes_lineatus_KJ966025.1     A..T........AT....T..T..G.....A........A..AC..T....T.....A..CT...
Agriotes_obscurus_KM442197.1     A..T........CT....T..C.....A..A........A..A........T.....A..CT...
PEI_1                            .................................................................
PEI_2                            .................................................................
PEI_3                            .................................................................
PEI_4                            .................................................................
PEI_5                            .................................................................
PEI_6                            .................................................................
PEI_7                            .................................................................
PEI_8                            .................................................................
PEI_9                            .................................................................
Truro_1                          .................................................................
Truro_2                          .................................................................
Truro_3                          .................................................................
Truro_4                          .................................................................
Truro_5                          .................................................................
Truro_6                          .................................................................
```

Figure S2. The multiple sequence alignment using ClustalW was created using the partial DNA sequences of the mt*COI* gene PCR products from wireworms collected from fields in Eastern Canada with three reference sequences from NCBI accession numbers KR124576.1 (*A. sputator*), KJ966025.1 (*A. lineatus*) and KM442197.1 (*A. obscurus*). Samples from PEI 1 - PEI 9 are wireworm individuals collected from the P.E.I. location; Truro 1 to Truro 6 were individuals collected from the Truro location. The dots represent the homologous base with the reference sequences, and the nucleotide changes are highlighted in the respective sequences.
